# Factors Affecting the Accuracy of Genomic Selection for Agricultural Economic Traits in Maize, Cattle, and Pig Populations

**DOI:** 10.3389/fgene.2019.00189

**Published:** 2019-03-14

**Authors:** Haohao Zhang, Lilin Yin, Meiyue Wang, Xiaohui Yuan, Xiaolei Liu

**Affiliations:** ^1^School of Computer Science and Technology, Wuhan University of Technology, Wuhan, China; ^2^Key Laboratory of Agricultural Animal Genetics, Breeding, and Reproduction of Ministry of Education and Key Laboratory of Swine Genetics and Breeding of Ministry of Agriculture, College of Animal Science and Veterinary Medicine, Huazhong Agricultural University, Wuhan, China; ^3^Department of Botany and Plant Sciences, University of California, Riverside, Riverside, CA, United States

**Keywords:** genomic prediction, marker density, BayesR, GBLUP, dominance

## Abstract

Genomic Selection (GS) has been proved to be a powerful tool for estimating genetic values in plant and livestock breeding. Newly developed sequencing technologies have dramatically reduced the cost of genotyping and significantly increased the scale of genotype data that used for GS. Meanwhile, state-of-the-art statistical methods were developed to make the best use of high marker density genotype data. In this study, 14 traits from four data sets of three species (maize, cattle, and pig) and five influential factors that affect the prediction accuracy were evaluated, including marker density (from 1 to ~600 k), statistical method (GBLUP-A, GBLUP-AD, and BayesR), minor allele frequency (MAF), heritability, and genetic architecture. Results indicate that in the GBLUP method, higher marker density leads to a higher prediction accuracy. In contrast, BayesR method needs more Monte Carlo Markov Chain (MCMC) iterations to reach the convergence and get reliable prediction values. BayesR outperforms GBLUP in predicting high or medium heritability trait that affected by one or several genes with large effects, while GBLUP performs similarly or slightly better than BayesR in predicting low heritability trait that controlled by a large amount of genes with minor effects. Prediction accuracy of trait with complex genetic architecture can be improved by increasing the marker density. Interestingly, for simple traits that controlled by one or several genes with large effects, higher marker density can cause a lower prediction accuracy if the QTN is included, but leads to a higher prediction accuracy if the QTN is excluded. The quantity of genetic markers with low MAF would not significantly affect the prediction accuracy of GBLUP, but results in a bad prediction accuracy performance of BayesR method. Compared with GBLUP-A, GBLUP-AD didn't show any advantages in capturing the non-additive variance for the traits with high heritability. The factors that affected prediction accuracy are discussed in this study and indicate that a combination of either GBLUP or BayesR method with moderate marker density and favorable polymorphism single nucleotide polymorphisms (SNPs) (~25 k SNPs) would always produce a good and stable prediction accuracy with acceptable breeding and computational costs.

## Introduction

The plant and animal breeding history experienced a long period of phenotypic selection and the genetic evolution progress was relative slow. In 1950s, Henderson developed Best Linear Unbiased Prediction(BLUP) method that can estimate the breeding value of each individual and had been widely used in livestock breeding (Henderson, 1975; Robinson, 1991; Jonas and de Koning, 2015). Meanwhile, in the plant breeding, breeders conduct well-designed trials to remove the environmental effects by planting the seeds repeatedly in multiple places and multiple years to estimate the breeding value without interference (Hickey et al., 2017). With development of genotyping technology, genetic markers that associated with breeding objective traits are used to assist selection (Lande and Thompson, 1990). However, target traits are always complex and controlled by genes with minor effects, which means a few markers explain limited genetic variance and contribute little to the genetic gain (Bernardo, 2008). In 2001, Meuwissen et al. proposed the concept of Genomic Selection (GS), which assumes that all genome segments contribute to the genetic variance and each segment is in high LD (Linkage Disequilibrium) with a minimum of one known genetic marker. Effects of all genetic markers, e.g., single nucleotide polymorphisms (SNPs), across the whole genome are estimated and used for predicting genetic merit of selection candidates (Meuwissen et al., 2001). The advances of high-throughput sequencing technologies have dramatically reduced the cost of genotyping, thus GS has been widely used in plant and animal breeding, and remarkably improves the selection accuracy as well as accelerates the breeding progress (Hayes et al., 2009a; Elshire et al., 2011; Bhat et al., 2016; Meuwissen et al., 2016; Crossa et al., 2017; Weller et al., 2017).

Statistical methods play important role in GS and can be classified into relationship-based methods and marker effect-based methods. Relationship-based method directly predicts breeding values using relationship information without estimating effects of genetic markers. Traditional BLUP method, which derives the relationship matrix from pedigree information, was widely used in livestock breeding (Hidalgo et al., 2015). However, the reproduction modes limit the usage of pedigree information based BLUP in plant breeding (Hickey et al., 2017). Compared with the traditional BLUP, Genomic BLUP (GBLUP) method, which uses a genomic information-based relationship matrix that provides more accurate relationship coefficients among individuals, increased the estimating accuracy of breeding values and was widely used in both plant and animal breeding (VanRaden, 2008). In order to maximize the use of pedigree information and genomic information, the single step BLUP method was developed and widely used in livestock breeding (Aguilar et al., 2010; Christensen and Lund, 2010; Christensen et al., 2012; Misztal et al., 2013; Zhang et al., 2016). Dominance effect was added in a multiple random effects model to capture the variance of non-additive genetic effect (Aliloo et al., 2017; Varona et al., 2018). Information from multi-omics, such as gene annotation and QTL (Quantitative Trait Loci) were used to weight genetic markers during the construction of the genomic relationship matrix used in GBLUP (Wimmer et al., 2013; An et al., 2017; Gao et al., 2017; Fikere et al., 2018; Schrag et al., 2018). Although a series of modified GBLUP methods have been developed to increase the prediction accuracy, the original GBLUP method, which is computationally efficient and meets the requirements of predicting breeding objective traits with complex genetic architecture, is still the most popular method applied in breeding practice.

Unlike GBLUP, marker effect-based methods have two major steps: (1) estimate the effects of genetic markers, and then (2) accumulate the effects to get the estimated breeding values (EBV) of each individual. The representative methods are ridge regression BLUP (RRBLUP) method and Bayesian alphabet methods (Whittaker et al., 1999; Endelman, 2011). RRBLUP assumes that all markers have the same genetic variance and has been proved to be equivalent with GBLUP method (Goddard, 2009; Hayes et al., 2009b). BayesA method assumes that variance of all marker effects follow an inverse-chi-square distribution (Meuwissen et al., 2001). In BayesB, only a proportion (Pi) of markers contribute to the target trait, and the variance of marker effects follow an inverse-chi-square distribution (Meuwissen et al., 2009). Later developed methods, such as BayesC, BayesCPi, BayesLASSO, made a better optimization of Pi and assigned distinct distributions for the variances of marker effects (Yi and Xu, 2008; Habier et al., 2011). Zhou et al. (2013) combined the hypothesis of both GBLUP and Bayesian methods and proposed a new hypothesis, assuming that all genetic markers have effects and the effects of a proportion of markers can be obtained by GBLUP while the other proportion of markers have an additional effect and the variance of the additional effects followed a normal distribution. The method is called BSLMM (Bayesian Sparse Linear Mixed Model) and the flexible hypothesis enables BSLMM achieve higher prediction accuracy than BayesCPi, BayesLASSO, and BVSR (Zhou et al., 2013). Moser et al. (2015) proposed BayesR method, which classified all markers into four groups and the variance of marker effects in different groups followed normal distributions with various variance categories (Moser et al., 2015). A mass of simulated and real experiments showed that BayesR always had higher or similar prediction accuracy as other marker effect based methods (Zeng and Zhou, 2017; Hayes et al., 2018).

Besides statistical method, the prediction accuracy may be also affected by marker density, minor allele frequency (MAF), heritability, and genetic architecture of target trait. Along with the development of cost-effective genotyping technology, high density marker genotype are used in GS and tell more information, this may increase the prediction accuracy (Elshire et al., 2011). However, the increased number of markers also drastically increase the computation burden, especially for the Bayesian alphabet methods because the number of unknown parameters that need to be estimated would greatly increase. The investigation of influences brought by statistical methods and marker densities on balancing the prediction accuracy and computational efficiency is necessary and important for practical breeding programs.

In this study, we investigated the prediction accuracies of 14 traits with various heritabilities and genetic architectures from two maize populations (Romay et al., 2013; Li et al., 2016; Liu et al., 2016), one cattle population (Matukumalli et al., 2009; Zhang et al., 2015), and one pig population. Potential GS-relevant factors, including marker density, statistical method, MAF, heritability, and genetic architecture were evaluated. A thorough understanding of GS-relevant factors would help breeders making a good breeding design to obtain reasonable genetic gain with reducing cost.

## Materials and Methods

### Genotypic and Phenotypic Data

Two maize populations investigated in this study are NAM_US and AMES populations. There are three flowering time traits in NAM_US population, including days to anthesis (DTA), days to silking (DTS), and anthesis-silking interval (ASI). All samples were planted under eight environments and DTA, DTS, and ASI were measured and calculated as described by Buckler et al. (2009). Samples without phenotypic records, SNPs with MAF <0.01 or in scaffold were removed in our former study (Li et al., 2016). A total of NAM_US 4,328 samples with 564,692 markers were used in this study. AMES is an inbred maize population and genotyped by GBS (Genotyping By Sequencing) (Romay et al., 2013; Liu et al., 2016). The AMES dataset includes 2,711 samples and 681,257 markers; three traits of the AMES population are growing degree DTS (GDD, 2,279 records), sweet vs. starchy kernel (SSK, 2,631 records), and yellow vs. white kernels (YWK, 1,595 records). SNPs with MAF <0.01 were removed in this study.

The cattle dataset contains 5,024 German Holstein bulls and all the bulls were genotyped using the Illumina Bovine SNP50 Beadchip (Matukumalli et al., 2009). Data quality control had been done in the previous study (Zhang et al., 2015) including the remove of SNPs with genotype call rate <95%, MAF <0.01 and SNPs failed to pass the Hardy-Weinberg Equilibrium (HWE) test with *p*-values < 10^−4^. A total of 42,551 SNPs were remained after the data quality control and used in this study. Estimated breeding values of three traits are provided to validate the GS-relevant factors: milk fat percentage (MFP), milk yield (MY), and somatic cell score (SCS). MFP, MY, and SCS are representatives for traits with different genetic architectures: (1) one gene with large effect (major gene) and a large number of genes with small effects (MFP), (2) several genes with moderate effects and many genes with small effects (MY), and (3) many genes with small effects (SCS).

The pig dataset is consisted of 3,534 animals from a nucleus pig farm of Pig Improvement Company (PIC) with five traits (Cleveland et al., 2012). All animals were genotyped by Illumina PorcineSNP60 chip, SNPs with HWE test *p-value* < 10^−4^, genotype call rate < 95%, and MAF < 0.03 were removed. For T1, T2, T3, T4, and T5, a total number of 43,428, 43,494, 43,407, 43,412, and 43,441 SNPs are remained in this study, respectively.

### Statistical Models for Genome-Enabled Predictions

As GBLUP is the most widely used statistical method in GS, BayesR is well-known for its high prediction accuracy and always has similar or higher prediction accuracy than other Bayesian alphabet methods (Moser et al., 2015; Zeng and Zhou, 2017). Three models were selected and tested in this study, including additive effect based GBLUP (GBLUP-A), additive and dominance effect based GBLUP (GBLUP-AD), and BayesR. GBLUP-A and GBLUP-AD methods were performed by HIBLUP software (https://hiblup.github.io/) and BayesR method was performed by BayesR software (http://cnsgenomics.com/software.html).

#### GBLUP-A Model

The GBLUP-A model can be described by the following equation:

$$y\;=\;Xb\;+\;Z_{a}u_{a}\;+\;e$$

where *y* is an *n* × 1 vector of observation, *b* is an *n* × *q* matrix of fixed effects, *u*_*a*_ is a *m* × 1 vector of breeding values and $u_{a}\sim MVN\left(0,G_{a}\sigma_{u_{a}}^{2}\right)$, *e* is an *n* × 1 vector of residuals and $e\sim MVN\left(0,I\sigma_{e}^{2}\right)$, *X* and *Z*_*a*_ are design matrices of *b* and *u*_*a*_, respectively. *n* represents sample size, *m* is the number of marker, *MVN* denotes multivariate normal distribution, *I* is an *n* × *n* identity matrix, *G*_*a*_ is additive effects based genomic relationship matrix of size *n* × *n*, and can be constructed by VanRaden method (VanRaden, 2008):

$$G_{a}=\frac{W_{a}W_{a}^{\prime }}{2\sum_{j=1}^{m}p_{j}\left(1-p_{j}\right)}$$

Where *W*_*a*_ is defined as:

$$W_{a}{}_{ij}=\{\begin{array}{cc} 2-2p_{j},\; & M_{ij}=AA\\ 1-2p_{j},\; & M_{ij}=AB\\ \;-2p_{j},\; & M_{ij}=BB \end{array}$$

Where *W*_*a*_*ij*__ represents the elements of *W*_*a*_ matrix at ith row and jth column, *M*_*ij*_ represents the combination of alleles at jth marker of ith individual, *p*_*j*_ is the allele frequency of A at jth marker.

#### GBLUP-AD Model

Additive and dominance effect based GBLUP model can be described by the following equation:

$$y\;=\;Xb\;+\;Z_{a}u_{a}\;+\;Z_{d}u_{d}+\;e$$

Where *y*, *Xb*, *Z*_*a*_*u*_*a*_, and *e* are the same as terms described in GBLUP-A model. Similar as *u*_*a*_,*u*_*d*_ is a *m* × 1 vector of dominance effect values and $u_{d}\sim MVN\left(0,G_{d}\sigma_{u_{d}}^{2}\right)$, *G*_*d*_ is dominance effect based genomic relationship matrix of size *n* × *n*, and can be constructed by following equation (Aliloo et al., 2017):

$$G_{d}=\frac{W_{d}W_{d}^{\prime }}{4\sum_{j=1}^{m}{\left(p_{j}\left(1-p_{j}\right)\right)}^{2}}$$

Where *W*_*d*_ is defined as:

$$W_{d}{}_{ij}=\{\begin{array}{cc} -2{\left(1-p_{j}\right)}^{2},\; & M_{ij}=AA\\ 2p_{j}\left(1-p_{j}\right),\; & M_{ij}=AB\\ \;-2p_{j}^{2},\; & M_{ij}=BB \end{array}$$

Where *W*_*dij*_ represents the elements of *W*_*d*_ matrix at ith row and jth column, *M*_*ij*_ and *p*_*j*_ are the same as described in GBLUP-A model.

#### BayesR Model

The statistical model of BayesR could be written as:

y = Xb + Zg + e

Where *y*, *Xb*, and *e* are the same as terms described in GBLUP-A model; *Z* is an *n* by m matrix of genotypes encoded as 0, 1, 2 copies of the reference allele; *g* is the sum of m-dimensional vector of SNP effects that derived from four independent normal distributions with mean of zero, and the relative variance for each distribution is fixed as:

$$\begin{array}{l} \rho \left(g|\pi ,\;\sigma_{g}^{2}\right)=\pi_{1}\times N\left(0,0\times \sigma_{g}^{2}\right)+\pi_{2}\times N\left(0,10^{-4}\times \sigma_{g}^{2}\right)\\ \;+\pi_{3}\times N\left(0,10^{-3}\times \sigma_{g}^{2}\right)+\pi_{4}\times N\left(0,10^{-2}\times \sigma_{g}^{2}\right) \end{array}$$

Where $\sigma_{g}^{2}$ is the additive genetic variances explained by SNPs, and π_1_+π_2_+π_3_+π_4_ = 1. The unknown parameters (***b***, π, *g*, $\sigma_{g}^{2}$, $\sigma_{e}^{2}$) are obtained from a Gibbs scheme based MCMC iterations.

#### Prediction Accuracy Evaluation

In this study, 5-folder cross validation was used to evaluate the prediction performances of each method. Each dataset was randomly divided into five parts, four parts of them were used as training dataset and the left part of dataset was used for validation. The prediction accuracy was measured with Pearson's correlation coefficient based on 10 replicates of 5-folder cross validation for each trait. To sample genetic markers at each level of marker density, a random seed was set to ensure that markers from low-density dataset were included in the high-density marker dataset. E.g., AMES data has 633 k SNPs in total and a random seed is used to ensure that all of the selected 100 k markers were included in the selected 200 k markers.

## Results

### Estimation of Heritability

We used both GBLUP-A model and GBLUP-AD model to estimate heritabilities by a cross validation procedure described in Prediction accuracy evaluation. For each population, the mean and standard error of heritabilities that estimated by 10 replicates of 5-folder cross validations are calculated (Table 1). Original phenotypic observations of two maize populations (AMES and NAM_US) are recorded in eight environments, and the corrected values of six traits which eliminate the environmental effect are used for heritability estimation. The heritabilities of six maize traits are high and in the range of between 0.64 and 0.97 and the dominance effect explained very little variance. The five traits of pig population have medium or low heritabilities, and the heritabilities estimated by GBLUP-A model and GBLUP-AD model are 0.03~0.37 and 0.04~0.42, respectively. The GBLUP-AD model explained about 10% more variances than GBLUP-A model. Since only EBVs are available in the cattle population, we do not estimate heritabilities.

**Table 1:** Heritability estimation in four populations.

**Data sets**	**Type of dependent variable**	**Traits**	**Heritability (standard error)**			
			**GBLUP-A**	**GBLUP-AD (ALL)**	**GBLUP-AD (A)**	**GBLUP-AD (D)**
AMES	Corrected Phenotypic observations	GDD	0.954 (0.0023)	0.951 (0.0025)	0.951 (0.0025)	0 (0)
		SSK	0.646 (0.0124)	0.662 (0.027)	0.662 (0.027)	0 (0)
		YWK	0.974 (0.0035)	0.978 (0.003)	0.863 (0.0351)	0.115 (0.0374)
NAM_US	Corrected Phenotypic observations	ASI	0.743 (0.0025)	0.758 (0.002)	0.758 (0.002)	0 (0)
		DTA	0.872 (0.0022)	0.872 (0.0022)	0.87 (0.0022)	0.002 (0.0009)
		DTS	0.876 (0.002)	0.876 (0.0019)	0.875 (0.0019)	0.002 (0.0009)
Pig_PIC	Phenotypic observations	T1	0.036 (0.003)	0.041 (0.0031)	0.036 (0.0036)	0.006 (0.0037)
		T2	0.283 (0.0041)	0.301 (0.0047)	0.272 (0.0053)	0.029 (0.0063)
		T3	0.206 (0.0054)	0.239 (0.0073)	0.186 (0.0061)	0.052 (0.0087)
		T4	0.332 (0.0036)	0.352 (0.005)	0.321 (0.0036)	0.031 (0.0043)
		T5	0.373 (0.0035)	0.421 (0.0067)	0.352 (0.0034)	0.069 (0.0069)
Cattle	EBVs	MFP	NA	NA	NA	NA
		MY	NA	NA	NA	NA
		SCS	NA	NA	NA	NA

In this study, prediction accuracy is defined as the Pearson correlation coefficient between the predicted individual's EBVs and their actual phenotypic records. As the EBVs that derived by BLUP method are unbiased estimation for phenotypic values (details see Supplementary 5), the expectation of the prediction accuracy should be equal to the square root of heritability and can be derived by following equations:

$$\begin{array}{l} Cor\left(EBV,y\right)\;=\frac{Cov\left(EBV,\;y\right)}{\sigma_{EBV}\sigma_{y}}\;\\ \;=\frac{Cov\left(EBV,\;EBV\right)+Cov\left(EBV,e\right)}{\sigma_{EBV}\sigma_{y}}\;\\ \;=\frac{\sigma_{EBV}^{2}}{\sigma_{EBV}\sigma_{y}}+\frac{Cov\left(EBV,e\right)}{\sigma_{EBV}\sigma_{y}}\;\\ \;=\sqrt{h^{2}}+0\;\\ \;=h \end{array}$$

Where *y* and *e* are the same as terms described in GBLUP-A model, *h*^2^ is heritability. From the evaluation results of 14 traits, we observed that the prediction accuracy of SSK is 10% higher than what we expected. SSK trait in AMES population is a case-control trait with skewed distribution. There are two potential reasons cause the prediction accuracy higher than *h*. One is that the covariance between EBV and residual in the actual data is not exactly equal to zero but a small number that is very close to zero. Additionally, phenotypic variance of SSK is around 0.01, both σ_*EBV*_ and σ_*y*_ are very small. Therefore, the ratio of *Cov*(*EBV, e*) and σ_*EBV*_σ_*y*_ is amplified. The other reason is that the skewed phenotype distribution of SSK easily introduced a large sampling error in the cross-validation processes.

### Effects of Marker Density, Statistical Method, and Genetic Architecture

**Figure 1:**
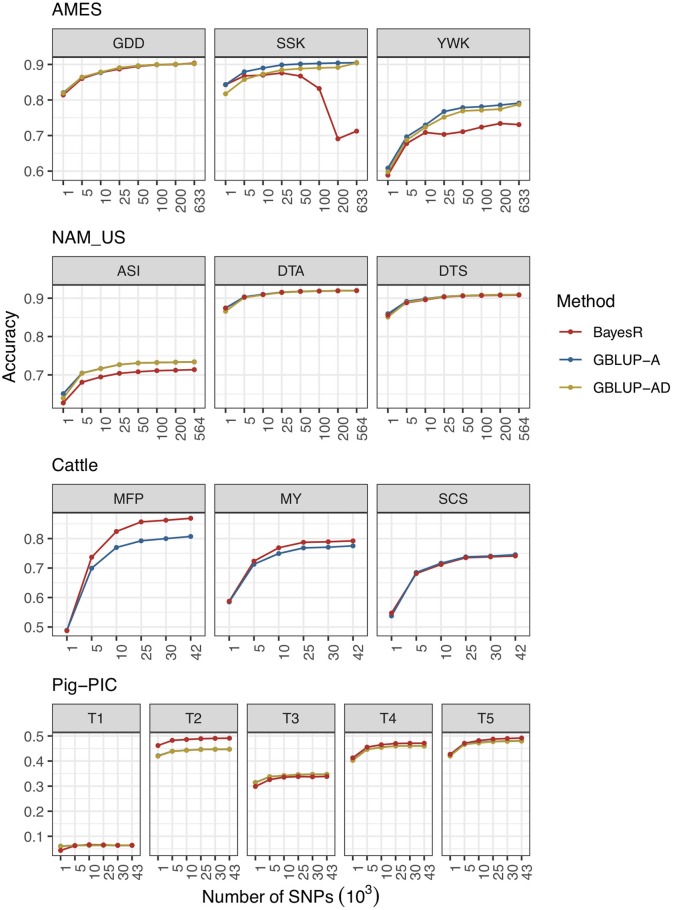
Prediction accuracies of combinations of different level of marker densities and methods in four populations.

In this section, various marker densities are designed to evaluate the prediction performances in AMES, NAM_US, Cattle and Pig-PIC populations. The results are shown in Figure 1, and the detailed information can be found in the Supplementary Table 1.

#### AMES

Three traits of AMES dataset are GDD, SSK, and YWK. All three traits have very high prediction accuracies because of the high heritabilities. Among three traits, the genetic architecture of GDD is relatively complicated and controlled by a big amount of genes. The prediction accuracy of GDD is increased with marker density in all three methods.

Unlike GDD, both SSK and YWK are affected by one or two genes with very big effect. The prediction accuracies of SSK and YWK are increased with marker density when using GBLUP-A and GBLUP-AD methods. However, as the marker density increases, the prediction accuracy of BayesR method fluctuates up and down and not performs as well as GBLUP methods. One potential reason is that prediction accuracy of BayesR is limited by the premature convergence of MCMC iterations. The total number of MCMC iterations is set to 200,000 and BayesR needs a much more MCMC iterations to reach the convergence. The prediction accuracy of the SSK trait is improved with the increased number of maximum MCMC iterations and more detailed information can be found in Supplementary Table 2 and Supplementary Figure 1. The other important reason is that the hypothesis of BayesR method is more adapted to complex traits that affected by four groups of genes mentioned before with effects from multiple categories but not well-adapted for the traits controlled by one or two genes with big effects.

#### NAM_US

All three traits in NAM population have high heritabilities and complex genetic architectures. The hypothesis of GBLUP model fits the complex traits well, GBLUP-A and GBLUP-AD perform better than BayesR in ASI trait and three methods perform similarly in DTA and DTS traits. For all three traits, prediction accuracies of all methods increased with marker density.

#### Cattle

Three traits in cattle population represent three distinct types of traits. MFP has high heritability and controlled by one major gene (DGAT1, Diacylglycerol acyltransferase 1 gene) and plenty of genes with minor effects; MY has medium heritability and also controlled by DGAT1 gene and some genes with medium and minor effects; SCS is a low heritability trait and controlled by a big amount of genes with minor effects. As the marker density of cattle data is moderate, the MCMC iterations reached convergence in all 10 replicates of cross validation procedure. The results indicate that BayesR outperforms GBLUP in the traits of MFP and MY and performs similarly with GBLUP in SCS trait. Prediction accuracies of three methods are increased with marker density in all three traits.

#### Pig-PIC

Five traits in pig population of PIC have medium or low heritabilities. BayesR performs slightly better than GBLUP in predicting most traits and shows more advantages in T2 as it is controlled by several genes with big effects. Prediction accuracy slightly increased with marker density for each trait.

### Prediction Accuracies of Traits With Simple Genetic Architectures

**Figure 2:**
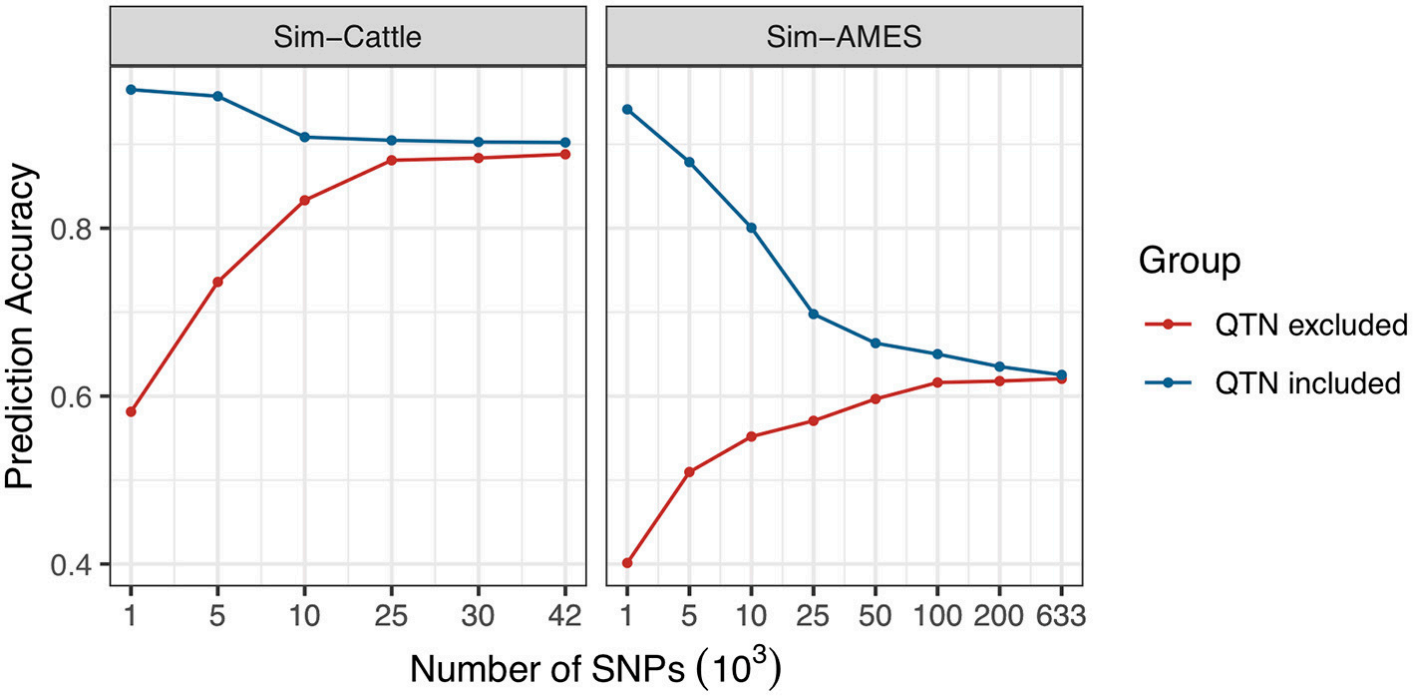
Effect of marker density on the prediction accuracies of traits with simple genetic architectures.

Genetic architectures of SSK, YWK, and MFP traits are relatively simple and controlled by one or several genes with very large effects. However, the prediction accuracies of the “simple” traits are increased with marker density as well and this doesn't comply with common sense. To find out the reason, we simulated two traits for AMES and cattle populations with heritability equals one and only controlled by one QTN. Two scenarios were simulated, including “QTN included” and “QTN excluded.” The results are shown in Figure 2, and the detailed information is provided in the Supplementary Table 3. Similar phenomena of prediction accuracy changes have been observed in both AMES and cattle populations. For “QTN included” scenario, QTN was included in the genotype data with different marker densities. With the increasing marker density, prediction accuracy continuously decreased. However, for “QTN excluded” scenario, the simulated QTN was removed from genotype and the prediction accuracy was increased with marker density. Using all available markers, prediction accuracies of both scenarios were finally stabilized at a certain level.

### Prediction Accuracies Affected by MAF

All of SSK, YWK, and MFP traits are affected by large effect genes and the hypothesis of BayesR is better suited for the genetic architectures of three traits than GBLUP. But BayesR only outperforms GBLUP-A in MFP trait while GBLUP-A performs better in SSK and YWK traits. Higher marker density causes MCMC iterations hard to converge, and this results BayesR a bad performance in prediction accuracy. However, for SSK trait, when the maximum number of MCMC iterations was more than 300 k, the prediction accuracy became steady and it was still not comparable with GBLUP. Therefore, there may be some other factors affected the prediction accuracy of BayesR method.

**Figure 3:**
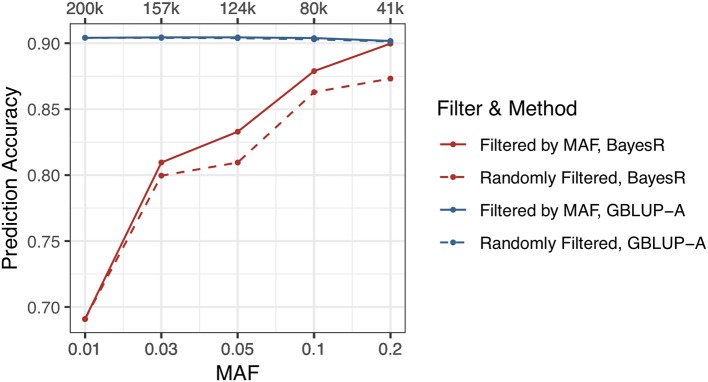
Effect of MAF on the prediction accuracies.

If there are plenty of genetic markers with low MAF included in the genotype data, more mistakes would be produced during evaluating the marker effects. SSK trait and 200 k genetic markers that have the lowest prediction accuracy were selected for this study. Genetic markers were filtered by five MAF thresholds, including 0.01, 0.03, 0.05, 0.1, 0.2, and the same amount of randomly selected genetic markers were also used in this study for comparison. The maximum number of MCMC iterations and burn-in iterations were set to 200/50 k. The results indicated that the MAF significantly affected the prediction accuracy and low MAF genetic markers would reduce the prediction accuracy performance of BayesR method. Filtered SNPs by MAF <0.2, BayesR method reaches the same prediction accuracy as GBLUP. In contrast, prediction accuracy of GBLUP was slightly affected by the low MAF genetic markers (Figure 3, Supplementary Table 4).

## Discussion

Previous studies indicate that prediction accuracy increases with marker density and a moderate marker density is always enough. Our results showed that prediction accuracy would continuously increase with marker density in GBLUP methods, especially when the marker density is low (e.g., <25 k SNPs). However, in the BayesR method, higher marker density requires much more MCMC iterations for the program to converge and get reliable prediction values. The default setting of BayesR parameters are 50,000 MCMC iterations with 20,000 burn-ins, this is usually enough for SNPs <50 k, but the risk of slow convergence or even no convergence increases with marker density.

Statistical methods significantly affect the prediction accuracy. Whittaker et al. assume that all genetic markers contribute to the trait and the effect follows a same distribution that all marker effects have the same variance, and the method is called Ridge Regression BLUP (RRBLUP) (Whittaker et al., 1999). Goddard (Goddard, 2009) and Hayes (Hayes et al., 2009a) proved that RRBLUP equals to GBLUP theoretically. However, the hypothesis limited the accuracy when predicting various types of traits with different genetic architectures. Therefore, a series of marker effect based methods, such as Bayesian methods [e.g., BayesR and Dirichlet Process Regression (Zeng and Zhou, 2017)] and machine learning methods [e.g., random forest and support vector machine (SVM) regression (de Oliveira et al., 2014)] were developed to resolve this limitation. Based on flexible hypothesizes of above methods, genetic markers can be classified into multiple groups and the marker effects are assumed to follow different distributions for each group; theoretically speaking, this helped to increase the prediction accuracy. Meanwhile, a series of relationship based methods were developed to improve the computational speed and prediction accuracy for association tests, and the related strategies can be also used for prediction, such as Factored Spectrally Transformed Linear Mixed Model (FaST-LMM) (Lippert et al., 2011), FaST-LMM-EWASher(Zou et al., 2014), Generalized Additive Model (GAM) (Jia et al., 2012), multikernel linear mixed model (MKLMM) (Weissbrod et al., 2016). With advanced Eigen decomposition method, FaST-LMM outperforms standard mixed linear model in terms of speed for association tests and the strategy can be also used to speed up GBLUP-A. FaST-LMM-EWASher model was developed to incorporate non-additive genetic effect and GAM utilized the pathway information and adjusted the gene length bias. Both strategies aimed to improve the prediction accuracy. MKLMM uses multiple-kernel machine learning approaches and incorporates genetic interactions to make the model adapted for complex traits and improve the prediction accuracy. Random forest and SVM regression methods can be also used to select trait favorite genetic markers for GBLUP methods and improve the prediction accuracy if there are genetic markers with big effects (Ogutu et al., 2011).

In this study, we selected a standard relationship based method (GBLUP) and a most commonly used marker effected based method (BayesR) for comparison. The results indicate that BayesR outperforms GBLUP for the trait with high or medium heritability and controlled by genes with large effects, such as the MFP trait in cattle data. However, in results of SSK and YWK traits that controlled by large effect genes, GBLUP performs better than BayesR. One reason is that the high marker density makes BayesR require more MCMC iterations. However, for SSK trait, when the maximum number of MCMC iterations is increased to more than 300 k, the prediction accuracy becomes steady and it is still not comparable with GBLUP. MAF significantly affected the prediction accuracy of marker effect based method—BayesR while slightly affected on the relationship based method—GBLUP. The results in Figure 3 indicate that including more genetic markers with lower MAF will cause a bad prediction accuracy performance of BayesR method. However, it is hard to say that genetic markers should be filtered by MAF before performing genomic prediction by using BayesR method, and the effect of MAF is always affected by the population size and phenotypic distribution. A combination of low MAF, small population size and skewed phenotypic distribution will lead to a bad performance. GBLUP performs similarly or slightly better than BayesR for the trait with low heritability and controlled by genes with minor effects, such as the T3 trait in pig data. GBLUP-AD model didn't show superiority in capturing non-additive effects for the high heritability traits under investigation, one possible reason is that most of traits in this study have high heritabilities while the traits with low heritabilities are always influenced more by dominance effects.

Not surprisingly, traits with higher heritability have higher prediction accuracy. Interestingly, we find that the increased marker density significantly affects the prediction accuracy of traits with simple genetic architecture that affected by one or several genes with large effects. As we know, the DGAT1 gene significantly affects MFP trait in cattle and the YWK trait is controlled by Y1 gene in AMES maize data (Buckner et al., 1990; Grisart et al., 2004). The marker density affects more on the prediction accuracy of MFP trait and YWK trait than on the traits not controlled by large effect genes.

The results indicate that prediction accuracy of trait with complex genetic architecture can be improved by increased marker density. But usually, the accuracy may decrease with the marker density when predicting a simple trait that controlled by single gene or several genes and this is different from the accuracy results of SSK, YWK, and MFP. In order to find out the reason, we simulated a trait that controlled by one QTN and explained all phenotypic variance; the prediction accuracy is expected to equal 100% when the QTN is included in GS model. When the information of more genetic markers was included in the model, the prediction accuracy will be decreased to the heritability divided by the total number of genetic markers, assuming all genetic markers are independent. Similar phenomenon had been observed in the real data analysis of MFP trait and YWK trait. When the QTN is removed from the genotype data, the prediction accuracy of the simulated trait increases with the marker density. The possible reason is that when QTN is excluded in genotype data, the prediction accuracy will be mainly affected by the genetic marker *G*_*m*_, which has the strongest linkage with QTN, as well as the number of genetic markers that are linked with QTN. The increased marker density will greatly increase the correlation between genetic marker *G*_*m*_ and QTN. As the marker density keeps increasing, the correlation between genetic marker *G*_*m*_ and QTN will become constant and prediction accuracy will be finally stabilized at a certain level.

## Conclusions

Genomic selection assisted breeding has been widely used in livestock breeding and plant breeding during the past decade. The strategy significantly increased the genetic gain by improving the accuracy of EBVs and accelerated the breeding cycles. In this study, we evaluated five influential factors of EBVs' prediction accuracy, including marker density, statistical method, MAF, heritability, and genetic architecture. Our results indicate that: (1) Increased marker density leads to a higher prediction accuracy of GBLUP methods, meanwhile, it also increases the risk of slow convergence and no convergence of MCMC iterations in BayesR method and this may result in a low prediction accuracy; (2) Statistical methods have their advantages when the genetic architectures of traits being predicting meet the hypothesis. BayesR outperforms GBLUP for the trait with high heritability and affected by one or several genes with large effects, while GBLUP performs similar or slightly better than BayesR for the trait with lower heritability and controlled by a big amount of genes with minor effects; (3) Prediction accuracy of trait with complex genetic architecture can be improved by increasing marker density. For simple traits that controlled by one or several genes with large effects, higher marker density can also lead to a higher prediction accuracy if the QTN is unknown and not included; (4) Including low MAF genetic markers would cause a bad performance of prediction accuracy when using BayesR method while GBLUP method has more tolerance and affected little by the MAF; (5) As most of the tested traits in this study have high heritabilities, GBLUP-AD performs similarly with GBLUP-A and doesn't show any advantages by capturing the non-additive variance. The understanding of GS related factors may help the breeders to design a more powerful and efficient genomic breeding plan. A combination of either GBLUP or BayesR method with moderate density (~25 k) and favorable polymorphism SNPs would always generate a good as well as stable prediction accuracy with acceptable breeding and computational costs.

## Data Availability

Publicly available datasets were analyzed in this study. The datasets can be found at https://onlinelibrary.wiley.com/doi/full/10.1111/tpj.13174 (maize-NAM_US), https://genomebiology.biomedcentral.com/articles/10.1186/gb-2013-14-6-r55 (maize-AMES), http://www.g3journal.org/content/5/4/615.long (cattle), and https://www.ncbi.nlm.nih.gov/pmc/articles/PMC3337471/bin/supp_2_4_429__index.html (pig).

## Author Contributions

XL, HZ, and XY conceived the study. HZ and LY wrote the codes and did the data analysis. XL, MW, HZ, and LY drafted the manuscript and all authors contributed to finalizing the writing.

### Conflict of Interest Statement

The authors declare that the research was conducted in the absence of any commercial or financial relationships that could be construed as a potential conflict of interest.
