# Supplementary material for: Factors Affecting the Accuracy of Genomic Selection for Agricultural Economic Traits in Maize, Cattle, and Pig Populations
Source: Front Genet. 2019 Mar 14;10:189. doi: 10.3389/fgene.2019.00189 (PMC6426750; doi:10.3389/fgene.2019.00189)
Supplement: Supplementary file 1 [file Data_Sheet_1.pdf]

Supplementary Material

1 Prediction accuracies of combinations of different level of marker densities and methods in four populations

Data Sets	Traits	SNP(k)	BayesR		GBLUP-A		GBLUP-AD	
			Mean	SE	Mean	SE	Mean	SE
AMES	GDD	1	0.81418643	0.00759488	0.82051776	0.00714996	0.81933354	0.00767545
AMES	SSK	1	0.84290389	0.01142008	0.84341125	0.01108595	0.81736082	0.03073252
AMES	YWK	1	0.58868695	0.01930262	0.60832792	0.03346189	0.59800502	0.03011935
AMES	GDD	5	0.86036927	0.00309464	0.86425389	0.00401998	0.8641124	0.00431263
AMES	SSK	5	0.86767804	0.01019821	0.87928526	0.00946658	0.85767382	0.02558474
AMES	YWK	5	0.67755261	0.0094952	0.69644458	0.02588635	0.68703003	0.0207413
AMES	GDD	10	0.87715204	0.00355679	0.87720203	0.00313362	0.87847026	0.00321788
AMES	SSK	10	0.86985258	0.01060978	0.88982125	0.00960477	0.8730259	0.02072332
AMES	YWK	10	0.70856989	0.01049775	0.7295223	0.02371917	0.72308356	0.0191757
AMES	GDD	25	0.88699196	0.00302425	0.8905514	0.00291343	0.89071705	0.00292782
AMES	SSK	25	0.87591194	0.00982414	0.89859505	0.00973782	0.88438235	0.01818934
AMES	YWK	25	0.70321578	0.01910665	0.76753859	0.02119661	0.75168312	0.01842612
AMES	GDD	50	0.89449796	0.00284201	0.89623778	0.00265405	0.89629489	0.00264779
AMES	SSK	50	0.86726748	0.01166124	0.90129041	0.00955717	0.8882266	0.01680364
AMES	YWK	50	0.71081662	0.02140028	0.77854267	0.02041715	0.76914895	0.01645513
AMES	GDD	100	0.89898599	0.00306407	0.89947317	0.00246793	0.89949978	0.0024625
AMES	SSK	100	0.8321209	0.01655667	0.90280727	0.00979812	0.89014767	0.01648946
AMES	YWK	100	0.72363293	0.02224831	0.78123454	0.02047518	0.77144619	0.01641651
AMES	GDD	200	0.89983172	0.00320658	0.90054407	0.00212909	0.90052561	0.00214195
AMES	SSK	200	0.69087842	0.05755389	0.90413902	0.00978726	0.89140555	0.01657876
AMES	YWK	200	0.73389633	0.0118234	0.78564059	0.01996015	0.77431815	0.01566385
AMES	GDD	633	0.90372323	0.00245757	0.902107	0.00202294	0.90209765	0.00202103
AMES	SSK	633	0.71244857	0.03867554	0.90478876	0.00971092	0.90401201	0.00970073
AMES	YWK	633	0.73073729	0.01391869	0.79112542	0.01930528	0.78738007	0.01941603
Cattle	MY	1	0.58753341	0.00413126	0.58522058	0.00362999	NA	NA
Cattle	MFP	1	0.48747037	0.01525321	0.48883465	0.01554239	NA	NA
Cattle	SCS	1	0.54733002	0.0043104	0.53760159	0.01089171	NA	NA
Cattle	MY	5	0.72346647	0.00684671	0.71285002	0.00539088	NA	NA
Cattle	MFP	5	0.73678145	0.0183879	0.69938205	0.01734829	NA	NA

Cattle	SCS	5	0.68150039	0.00254407	0.68489337	0.00465379	NA	NA
Cattle	MY	10	0.76874008	0.00452376	0.74912699	0.0041657	NA	NA
Cattle	MFP	10	0.82405285	0.00607785	0.76975921	0.00735844	NA	NA
Cattle	SCS	10	0.71209311	0.00306925	0.71635073	0.00721302	NA	NA
Cattle	MY	25	0.78733173	0.00361454	0.76828894	0.00355269	NA	NA
Cattle	MFP	25	0.85661435	0.00206862	0.79240098	0.00546608	NA	NA
Cattle	SCS	25	0.73514357	0.00315351	0.7377974	0.00617188	NA	NA
Cattle	MY	30	0.78910595	0.00374984	0.770721	0.00309295	NA	NA
Cattle	MFP	30	0.86214654	0.00222038	0.79987825	0.00512041	NA	NA
Cattle	SCS	30	0.73801404	0.00301559	0.7404855	0.00658317	NA	NA
Cattle	MY	42	0.79225101	0.00366746	0.77524638	0.00294305	NA	NA
Cattle	MFP	42	0.86881505	0.00262009	0.80723309	0.00532878	NA	NA
Cattle	SCS	42	0.7411081	0.00280782	0.74522676	0.00727193	NA	NA
NAM_US	DTA	1	0.87426928	0.00265104	0.87406472	0.0024762	0.86585825	0.00276805
NAM_US	DTS	1	0.85591707	0.00320012	0.85933664	0.00457071	0.85080096	0.00502874
NAM_US	ASI	1	0.62692935	0.00535896	0.65099485	0.02388839	0.63957428	0.02460301
NAM_US	DTA	5	0.90279697	0.00171344	0.90347262	0.00157442	0.90158567	0.00159263
NAM_US	DTS	5	0.88805951	0.00201977	0.89181301	0.00282688	0.88960905	0.00288535
NAM_US	ASI	5	0.68086486	0.00422468	0.7046163	0.02044188	0.7038785	0.02031267
NAM_US	DTA	10	0.90954941	0.00141041	0.9099666	0.00116587	0.90862801	0.00128878
NAM_US	DTS	10	0.89581858	0.00194667	0.89831299	0.0022945	0.8967635	0.00248832
NAM_US	ASI	10	0.69457579	0.00312945	0.71647015	0.01928542	0.71615163	0.01921734
NAM_US	DTA	25	0.91536994	0.0012205	0.91501945	0.00101561	0.91478721	0.00106775
NAM_US	DTS	25	0.90323127	0.00168918	0.90448986	0.00186468	0.90431865	0.00192631
NAM_US	ASI	25	0.70403488	0.00367015	0.72678125	0.01872061	0.72674804	0.01871818
NAM_US	DTA	50	0.91777818	0.00128211	0.91751332	0.00112138	0.91745173	0.00115921
NAM_US	DTS	50	0.90585141	0.00165758	0.90666949	0.00189515	0.90658152	0.00190307
NAM_US	ASI	50	0.70808407	0.00344548	0.730973	0.018297	0.7309987	0.01828994
NAM_US	DTA	100	0.91849695	0.00135005	0.91861087	0.00117032	0.91861323	0.00119532
NAM_US	DTS	100	0.90691242	0.00173106	0.90800086	0.00193447	0.90798859	0.00193452
NAM_US	ASI	100	0.71100205	0.00296248	0.73212828	0.01845124	0.73213352	0.01844694
NAM_US	DTA	200	0.91931661	0.00133732	0.91928517	0.00110321	0.91929913	0.00112173
NAM_US	DTS	200	0.90767283	0.00176576	0.90890515	0.00196588	0.90877864	0.00198122
NAM_US	ASI	200	0.7121484	0.00319036	0.73307801	0.01851008	0.73309428	0.01850657
NAM_US	DTA	564	0.92003585	0.00112905	0.9196484	0.00114129	0.91964588	0.00114054
NAM_US	DTS	564	0.90805129	0.00177732	0.90919925	0.00198111	0.90919211	0.00198205
NAM_US	ASI	564	0.71367208	0.00329173	0.73388878	0.01845187	0.73391443	0.01844548
Pig-PIC	T1	1	0.04361301	0.01157807	0.06054901	0.00763078	0.05997783	0.00812815

Pig-PIC	T2	1	0.46178721	0.00519117	0.42087491	0.04031598	0.41953117	0.04106691
Pig-PIC	T3	1	0.29861788	0.01074579	0.31437515	0.01938465	0.31356158	0.0192477
Pig-PIC	T4	1	0.41279832	0.0081684	0.40324726	0.01023593	0.40254639	0.01022007
Pig-PIC	T5	1	0.4268313	0.00946759	0.42122211	0.00782363	0.42018141	0.00814701
Pig-PIC	T1	5	0.0624071	0.01189008	0.06338565	0.00849972	0.06329832	0.00874322
Pig-PIC	T2	5	0.48249505	0.00428494	0.43911863	0.04460313	0.43886228	0.04469716
Pig-PIC	T3	5	0.32591788	0.01158068	0.33767765	0.02032316	0.33746433	0.02031912
Pig-PIC	T4	5	0.45535167	0.00612027	0.44665222	0.01192064	0.44640421	0.01185304
Pig-PIC	T5	5	0.47094866	0.00760855	0.46715821	0.00670624	0.46625608	0.00721619
Pig-PIC	T1	10	0.0663917	0.01060171	0.06377413	0.00852224	0.0637651	0.00875014
Pig-PIC	T2	10	0.48590916	0.00491886	0.44318187	0.04520001	0.44290413	0.04527062
Pig-PIC	T3	10	0.33566658	0.01197914	0.34176793	0.02001904	0.34160671	0.02002632
Pig-PIC	T4	10	0.46496675	0.00591948	0.45480112	0.01168931	0.45438284	0.01166266
Pig-PIC	T5	10	0.48141524	0.00716032	0.47388309	0.00634623	0.47215803	0.0071122
Pig-PIC	T1	25	0.06561514	0.01184301	0.06424789	0.00963828	0.06412519	0.00977674
Pig-PIC	T2	25	0.48902659	0.00493239	0.44626673	0.04615443	0.44602392	0.04620703
Pig-PIC	T3	25	0.33806226	0.01021044	0.34572548	0.01942386	0.345452	0.01936453
Pig-PIC	T4	25	0.46994035	0.00594634	0.46010576	0.01212942	0.4597971	0.01222279
Pig-PIC	T5	25	0.48749375	0.00497797	0.47818274	0.00614488	0.47730505	0.00678229
Pig-PIC	T1	30	0.06369436	0.01090738	0.06414874	0.00977606	0.06424283	0.00994006
Pig-PIC	T2	30	0.49044261	0.00516771	0.44677705	0.04620948	0.4465579	0.04630452
Pig-PIC	T3	30	0.33722175	0.01034349	0.34692946	0.01960429	0.34673385	0.01953945
Pig-PIC	T4	30	0.47088766	0.00580037	0.4602381	0.0117673	0.4599771	0.01185815
Pig-PIC	T5	30	0.48985615	0.00536446	0.47949674	0.00622867	0.47867311	0.00682928
Pig-PIC	T1	43	0.06420437	0.01103123	0.06361972	0.00962685	0.06384845	0.00983455
Pig-PIC	T2	43	0.49109756	0.00495001	0.44709099	0.04609947	0.44684173	0.04614857
Pig-PIC	T3	43	0.33844857	0.0104758	0.34703134	0.01925494	0.34680666	0.01917676
Pig-PIC	T4	43	0.47108264	0.00582567	0.46003562	0.0120334	0.45982768	0.01218563
Pig-PIC	T5	43	0.49177605	0.00552517	0.48044955	0.00624048	0.47951399	0.00677525

Supplementary Table 1. Prediction accuracies on combinations of different levels of marker densities and methods for four populations. Mean and standard error (SE) of prediction accuracies from 10 replicated 5-folder cross validation are shown in the table. The results in this table are corresponding to Figure 1 in the manuscript.

2 Effect of maximum number of MCMC iterations on prediction accuracy of BayesR.

Data Sets	Traits	Method	Length of MCMC iterations(k)	Length of Burn-in iterations(k)	Replicate of 5-folder Cross validation	Seeds	Mean of Accuracies
AMES	SSK	BayesR	200	5	1	250	0.7652728
AMES	SSK	BayesR	200	5	2	134	0.7628464
AMES	SSK	BayesR	200	5	3	517	0.4617012
AMES	SSK	BayesR	200	5	4	160	0.6861156
AMES	SSK	BayesR	200	5	5	946	0.8020658
AMES	SSK	BayesR	200	5	6	250	0.7916131
AMES	SSK	BayesR	200	5	7	134	0.7365533
AMES	SSK	BayesR	200	5	8	517	0.2686448
AMES	SSK	BayesR	200	5	9	160	0.826424
AMES	SSK	BayesR	200	5	10	946	0.8075473
AMES	SSK	BayesR	250	6.25	1	250	0.7615041
AMES	SSK	BayesR	250	6.25	2	134	0.7495268
AMES	SSK	BayesR	250	6.25	3	517	0.6692939
AMES	SSK	BayesR	250	6.25	4	160	0.828367
AMES	SSK	BayesR	250	6.25	5	946	0.7959149
AMES	SSK	BayesR	250	6.25	6	250	0.7892819
AMES	SSK	BayesR	250	6.25	7	134	0.7466092
AMES	SSK	BayesR	250	6.25	8	517	0.5336708
AMES	SSK	BayesR	250	6.25	9	160	0.8109914
AMES	SSK	BayesR	250	6.25	10	946	0.8132789
AMES	SSK	BayesR	300	7.5	1	250	0.7574101
AMES	SSK	BayesR	300	7.5	2	134	0.7389148
AMES	SSK	BayesR	300	7.5	3	517	0.7227775
AMES	SSK	BayesR	300	7.5	4	160	0.8305681
AMES	SSK	BayesR	300	7.5	5	946	0.7942602

AMES	SSK	BayesR	300	7.5	6	250	0.7871148
AMES	SSK	BayesR	300	7.5	7	134	0.7371667
AMES	SSK	BayesR	300	7.5	8	517	0.7832897
AMES	SSK	BayesR	300	7.5	9	160	0.8036355
AMES	SSK	BayesR	300	7.5	10	946	0.8194018
AMES	SSK	BayesR	350	8.75	1	250	0.7522835
AMES	SSK	BayesR	350	8.75	2	134	0.7342791
AMES	SSK	BayesR	350	8.75	3	517	0.7176291
AMES	SSK	BayesR	350	8.75	4	160	0.8206466
AMES	SSK	BayesR	350	8.75	5	946	0.7930056
AMES	SSK	BayesR	350	8.75	6	250	0.7889083
AMES	SSK	BayesR	350	8.75	7	134	0.7323591
AMES	SSK	BayesR	350	8.75	8	517	0.8331317
AMES	SSK	BayesR	350	8.75	9	160	0.797782
AMES	SSK	BayesR	350	8.75	10	946	0.8256349
AMES	SSK	BayesR	400	10	1	250	0.7476947
AMES	SSK	BayesR	400	10	2	134	0.7357039
AMES	SSK	BayesR	400	10	3	517	0.7136265
AMES	SSK	BayesR	400	10	4	160	0.8127463
AMES	SSK	BayesR	400	10	5	946	0.7930056
AMES	SSK	BayesR	400	10	6	250	0.7893001
AMES	SSK	BayesR	400	10	7	134	0.7282351
AMES	SSK	BayesR	400	10	8	517	0.8288769
AMES	SSK	BayesR	400	10	9	160	0.7933899
AMES	SSK	BayesR	400	10	10	946	0.8292063

Supplementary Table 2. Effect of maximum number of MCMC iterations on prediction accuracy of BayesR.

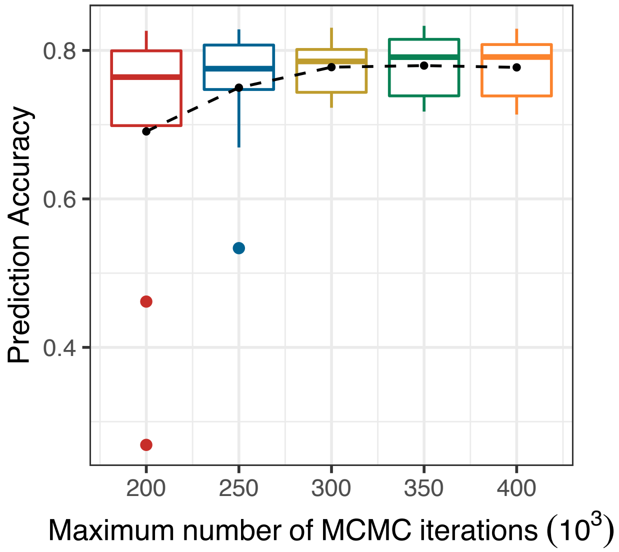

Supplementary Figure 1. Effect of maximum number of MCMC iterations on prediction accuracy of BayesR. Prediction accuracies of SSK trait were estimated by BayesR model with various sets of maximum MCMC iteration numbers. We randomly selected 200,000 SNPs for this study. The maximum number of MCMC iterations and the number of burn-in iterations were set to 200k/50k, 250k/62.5k, 300k/75k, 350k/87.5k, 400k/100k, respectively. The detailed results information are provided in the Supplementary Table 2. Along with the increase of maximum number of MCMC iterations and burn-in iterations, the prediction accuracy of BayesR increased. Compared with the set of 200k/50k, the prediction accuracy of 400k/100k improved more than 10%.

3 Effect of marker density on the prediction accuracies of traits with simple genetic architectures.

Data Sets	Group	SNP(k)	Method	Mean of Accuracies	SE of Accuracies
Sim-Cattle	QTN excluded	1	GBLUP-A	0.58142834	0.04111119
Sim-Cattle	QTN included	1	GBLUP-A	0.96513286	0.03626785
Sim-Cattle	QTN excluded	5	GBLUP-A	0.73604956	0.01289361
Sim-Cattle	QTN included	5	GBLUP-A	0.95721724	0.01986184
Sim-Cattle	QTN excluded	10	GBLUP-A	0.83328365	0.01012578
Sim-Cattle	QTN included	10	GBLUP-A	0.90858408	0.00703299
Sim-Cattle	QTN excluded	25	GBLUP-A	0.88095687	0.00554079
Sim-Cattle	QTN included	25	GBLUP-A	0.90471253	0.00446429
Sim-Cattle	QTN excluded	30	GBLUP-A	0.88366662	0.00374882
Sim-Cattle	QTN included	30	GBLUP-A	0.90273753	0.00384356
Sim-Cattle	QTN excluded	42	GBLUP-A	0.88808828	0.00248245
Sim-Cattle	QTN included	42	GBLUP-A	0.90218812	0.00232702
Sim-AMES	QTN excluded	1	GBLUP-A	0.40130205	0.01038206
Sim-AMES	QTN included	1	GBLUP-A	0.94160668	0.06124245
Sim-AMES	QTN excluded	5	GBLUP-A	0.5095827	0.01009804
Sim-AMES	QTN included	5	GBLUP-A	0.87883281	0.04117538
Sim-AMES	QTN excluded	10	GBLUP-A	0.55182287	0.0102861
Sim-AMES	QTN included	10	GBLUP-A	0.80056068	0.02980899
Sim-AMES	QTN excluded	25	GBLUP-A	0.57060054	0.01409398
Sim-AMES	QTN included	25	GBLUP-A	0.69776305	0.02131359
Sim-AMES	QTN excluded	50	GBLUP-A	0.59661025	0.01492414
Sim-AMES	QTN included	50	GBLUP-A	0.66311124	0.01795156
Sim-AMES	QTN excluded	100	GBLUP-A	0.61622204	0.01271243
Sim-AMES	QTN included	100	GBLUP-A	0.6500602	0.01295391
Sim-AMES	QTN excluded	200	GBLUP-A	0.61791859	0.01310678
Sim-AMES	QTN included	200	GBLUP-A	0.635113	0.0124621
Sim-AMES	QTN excluded	633	GBLUP-A	0.62065391	0.01382256
Sim-AMES	QTN included	633	GBLUP-A	0.62533146	0.01311782

Supplementary Table 3. Effect of marker density on the prediction accuracies of traits with simple genetic architectures. The data in this table are corresponding to Figure 3 in the manuscript.

4 Effect of MAF on the prediction accuracies.

Data Sets	Traits	MAF	SNP(k)	Filter Method	Method	Mean of Accuracies	SE of Accuracies
AMES	SSK	0.01	200	Filtered by MAF	BayesR	0.69087842	0.05755389
AMES	SSK	0.03	157	Filtered by MAF	BayesR	0.80957379	0.02199821
AMES	SSK	0.05	124	Filtered by MAF	BayesR	0.83290974	0.01840459
AMES	SSK	0.1	80	Filtered by MAF	BayesR	0.87886489	0.01419412
AMES	SSK	0.2	41	Filtered by MAF	BayesR	0.8997123	0.0097839
AMES	SSK	0.01	200	Randomly Filtered	BayesR	0.69087842	0.05755389
AMES	SSK	0.01	157	Randomly Filtered	BayesR	0.7995984	0.01485061
AMES	SSK	0.01	124	Randomly Filtered	BayesR	0.80954931	0.01383694
AMES	SSK	0.01	80	Randomly Filtered	BayesR	0.86291868	0.01188254
AMES	SSK	0.01	41	Randomly Filtered	BayesR	0.87319479	0.01185445
AMES	SSK	0.01	200	Filtered by MAF	GBLUP-A	0.90413902	0.00978726
AMES	SSK	0.03	157	Filtered by MAF	GBLUP-A	0.90444684	0.01029713
AMES	SSK	0.05	124	Filtered by MAF	GBLUP-A	0.90444621	0.01026337
AMES	SSK	0.1	80	Filtered by MAF	GBLUP-A	0.9039314	0.01016967
AMES	SSK	0.2	41	Filtered by MAF	GBLUP-A	0.90175487	0.0101909
AMES	SSK	0.01	200	Randomly Filtered	GBLUP-A	0.90413902	0.00978726
AMES	SSK	0.01	157	Randomly Filtered	GBLUP-A	0.90405365	0.01023856
AMES	SSK	0.01	124	Randomly Filtered	GBLUP-A	0.9038311	0.01027898
AMES	SSK	0.01	80	Randomly Filtered	GBLUP-A	0.90293217	0.01044674
AMES	SSK	0.01	41	Randomly Filtered	GBLUP-A	0.90134265	0.01044877

Supplementary Table 4. Effect of MAF on the prediction accuracies. The data in this table are corresponding to Figure 4 in the manuscript.

5 EBV that derived by BLUP method is unbiased.

Consider the following mixed linear model:

$$y = Xb + Zu + e$$

Where $u \sim MVN(0, \sigma_u^2 G)$, $e \sim MVN(0, \sigma_e^2 I)$, G is the genomic relationship matrix, I is the identity matrix, MVN denotes multivariate normal distribution. To find the best linear unbiased prediction of u denote by $\hat{u}$, there are 3 requirements: 1) $\hat{u}$ is a linear function of y; 2) $\hat{u}$ is unbiased for u and $E(\hat{u} - u) = 0$; 3) There are no other linear and unbiased predictors v to make $Var(v - u)$ less than $Var(\hat{u} - u)$. Using Henderson Method 3, $\hat{u}$ can be predicted by solving the following mixed model equation:

$$\begin{bmatrix} X'X & X'Z \\ Z'X & Z'Z + \lambda G^{-1} \end{bmatrix} \begin{bmatrix} \hat{b} \\ \hat{u} \end{bmatrix} = \begin{bmatrix} X'y \\ Z'y \end{bmatrix}$$

Where λ is the ratio between σ_e^2 and σ_g^2 .
